# Better cardiac care: health professional’s perspectives of the barriers and enablers of health communication and education with patients of Aboriginal and Torres Strait Islander descent

**DOI:** 10.1186/s12913-019-3917-4

**Published:** 2019-02-07

**Authors:** Jordan Stanford, Karen Charlton, Anne-Therese McMahon, Scott Winch

**Affiliations:** 1 School of Medicine, University of Wollongong, Wollongong, NSW 2522 Australia; 2 Illawarra Health and Medical Research Institute, Wollongong, NSW 2522 Australia; 3 School of Health & Society, University of Wollongong, Wollongong, NSW 2522 Australia

**Keywords:** Indigenous, Aboriginal, Health promotion, Cardiovascular health, Health education resources, Health service

## Abstract

**Background:**

A body of knowledge continues to grow regarding Aboriginal perspectives on current challenges and barriers to health literacy and access to health services. However, less is known from the perspectives of health professionals who work in cardiac care. Given their role in delivering patient education, health practitioners could provide useful insights into potential solutions to improve patient-practitioner communication. The primary aim was to explore perspectives of health professionals who work in coronary care units regarding the enablers, barriers and potential solutions for patient-practitioner communication with patients of Aboriginal and Torres Strait Islanders descent. The secondary aim was to evaluate the acceptability and value of two videos developed with key stakeholders to provide culturally appropriate education.

**Methods:**

Participants were recruited from two major regional hospitals. In-depth, semi-structured interviews were conducted with 17 health professionals (11 Nurses, five Cardiologists and one Aboriginal Health Worker). Interviews were recorded, de-identified and transcribed verbatim. Transcripts were analysed using constant comparison, interpreted through inductive thematic analysis and final themes were agreed through consensus with secondary researcher**.**

**Results:**

Health professionals acknowledged that existing barriers resulted from organisational structures entrenched in the healthcare system, impacted on the practitioners’ ability to provide culturally appropriate, patient-centred care. Lack of time, availability of culturally appropriate resources and the disconnection between Western medical and Aboriginal views of health were the most common challenges reported. The two videos evaluated as part of this study were found to be a useful addition to practice. Strengths in the videos design were the use of Aboriginal and Torres Strait Islander actors and positive messaging to convey health related topics. Further improvements included additional information related to common tests and procedures to allow for realistic expectations of patient care.

**Conclusion:**

Re-modelling of organisational structures is required in order to promote a more culturally-friendly and welcoming environment to encourage Aboriginal and Torres Strait Islanders to engage with mainstream cardiac care services. The videos that were developed using principles that are sensitive to Aboriginal health views, may offer an additional way in which to overcome existing barriers to effective patient-practitioner communication with Aboriginal and Torres Strait Islanders.

**Electronic supplementary material:**

The online version of this article (10.1186/s12913-019-3917-4) contains supplementary material, which is available to authorized users.

## Background

The state of Aboriginal health is well documented in the literature. It is known that people of the Aboriginal^1^ community suffer worse health issues than any other sector of the population in Australia [1]. Heart and circulatory conditions contribute most to the disease burden and life expectancy gap for Aboriginal and Torres Strait Islanders [2–5]. In addition, people of Aboriginal descent are almost five times more likely than non-Aboriginal people to be hospitalised for conditions that are potentially avoidable with preventive measures, including receiving appropriate healthcare advice and education [6]. A likely contributor to these higher rates of cardiac conditions, is the limited access and engagement in health services that aim to prevent and treat cardiac conditions for Aboriginal Australians [7]. Known barriers for Aboriginal Australians to access mainstream healthcare services are vast; some may include a fear of hospitalisation, lack of understanding of diagnostic and treatment pathways [8], and a lack of services available to meet their specific cultural needs [6, 9].

Hospitals and outpatient clinics tend to rely heavily on printed material to convey information, which may not be appropriate and act as a deterrent to some people of the Aboriginal community who have limited literacy skills, further heightening feelings of ‘shame’ and resulting in disengagement with the health service [8]. Electronic media such as videos on the other hand, often incorporate real-life situations [10], and offer a process that can be interactive and participatory, rather than rely on the passive transfer of information [11]. Educational videos are particularly useful in situations where shyness and embarrassment may inhibit direct discussions with healthcare practitioners, and redress balance if the quality of face-to-face communication or education is poor [12]. In addition, features of electronic, audiovisual or printed education materials could potentially address barriers previously discussed, such as the access to and reach of health information, as these resources are usually economically easier to produce, update and disseminate [13–17].

The Better Cardiac Care Initiative for Aboriginal and Torres Strait Islander people is a National project supported by the Australian Health Ministers’ Advisory Council [5, 18]. This joint State and Federal effort aims to reduce mortality and morbidity from cardiac health conditions by increasing access to services, ensure better management of risk factors and treatment options, and improve the coordination of care [5]. Within this initiative, the New South Wales Ministry of Health (NSW MoH) developed a series of four culturally specific, evidence based educational videos to form part of a broader package to improve cardiac care [19] and enhance current face-to-face education consultations between health professionals and patients in clinical and community settings. The videos were developed through collaboration between the NSW MoH and a range of partners including the Better Cardiac Care Aboriginal Advisory Group, the National Heart Foundation, NSW Ambulance service and clinicians from Aboriginal Medical Services and Local Health Districts (LHDs). The videos were produced by an Australian Aboriginal filming company, featured Aboriginal community members and professional actors, and the script was developed using principles of educational materials that are known to drive behaviour change in Indigenous populations [20].

While the current body of knowledge continues to grow regarding Aboriginal perspectives on challenges and barriers in health literacy and access to health services [21], little is known from the perspectives of health professionals working with Aboriginal patients. Given their experience and central role in the delivery of patient education, health professionals could provide useful knowledge and insight into potential solutions to current health education practice and address potential barriers required for uptake. Further, successful implementation of health education resources in clinical practice requires the support and acceptance from health professionals [22].

This study was conducted to address this gap. The primary aim was to explore perspectives of health professionals who work in inpatient or outpatient cardiac or coronary care units regarding the enablers, barriers and potential solutions for patient-practitioner communication and education with Aboriginal and Torres Strait Islanders. A secondary aim was to evaluate the acceptability and value of two Better Cardiac Care videos for use in clinical practice.

## Methods

### Recruitment, screening and enrolment

Participants were recruited from two major regional hospitals in New South Wales, Australia. A purposive sampling approach was used to recruit participants from part of the target demographic of interest as recommended by Harris et al [23]. The inclusion criteria were health professionals who work in inpatient or outpatient cardiac and coronary care units, and employed by the LHD where ethics was granted. Targeted health disciplines included Nurse Practitioners, Doctors, Allied Health staff, and Aboriginal Liaison Officers, all who play a major role in the clinical management and education of patients. Health professionals who worked outside areas relevant to cardiac care were excluded, which was naturally achieved through the recruitment design.

Recruitment was conducted through multi­disciplinary meetings in which a 10-min presentation about the study was provided. Interested participants were encouraged to leave their details to be followed up by phone or email in order to provide further details regarding the study, confirm enrolment and schedule an interview. In addition, email invitations were sent to key stakeholders who were identified by the Heads of the Cardiology Department in both hospitals. A snowballing sampling approach was also utilised as recommended by Harris [23] to extend the purposive sample. These additional participants were identified with the assistance of enrolled participants (Cardiac Rehabilitation co-ordinators and a Coronary CNC) to enable the inclusion of other key medical and allied health colleagues within their unit.

### Data collection and analysis

Each interview was conducted by a single moderator [JS] using a semi-structured script (Item S1, Additional file 1). All interview materials including the semi-structured guide and demographic questionnaire (Item S2, Additional file 1) were designed to encompass key areas of importance identified from the literature [24] and were reviewed by all members of the research team for quality purposes. The Aboriginal member of the research team specifically reviewed the content of materials to ensure they were culturally appropriate. The interviews took place between June and August 2016 and ran for 30–40 min each. All interviews were held in secluded rooms separate from the hospital wards or outpatient clinics. To satisfy the secondary aim of this study, additional time was allowed for the participants to view and comment on two of the four videos in the series, ‘*Aunty Gloria’s Story: At the Hospital’* and ‘*Aunty Gloria’s Story: Keeping your heart health’* [19, 25, 26]. The interviewer kept field notes summarising each interview, which included observed non-verbal communication to enrich the final analysis [27]. Exploration of new concepts offered by the participant were followed up, and further clarification of unclear concepts was completed [24]. Theoretical saturation of themes was reached by the twelfth interview.

The recordings were transcribed by an external provider (*Digitype)* and assessed for accuracy against all the digital recordings by a member of the research team [JS]. A systematic approach using content and inductive thematic analysis were employed to elicit explicit meaning obtained from the verified transcribed data set [28]. The coder [JS] carried out an initial qualitative analysis by coding all written transcripts by hand to develop categories and begin to formulate larger themes following the Braun and Clarke approach [29] to enable full data immersion, code identification, and inductive thematic analysis. Analysis was supported by Nvivo 11 software for Mac (QSR International Pty Ltd., Melbourne, Australia, 2014), to enable review of coded categories, deviant cases and thematic hierarchies as well as ensure that all the data was included into the analysis [30]. Inter-reliability of codes were checked in Nvivo and reviewed by a secondary member of the research team [AM] to ensure credibility of coding analysis [23, 31]. Finally, concept maps were constructed which assisted in the identification of further unique categories for deeper exploration [28]. Archetypical quotes were also identified to authenticate final themes with a secondary researcher [AM].

## Results

**Fig. 1:**
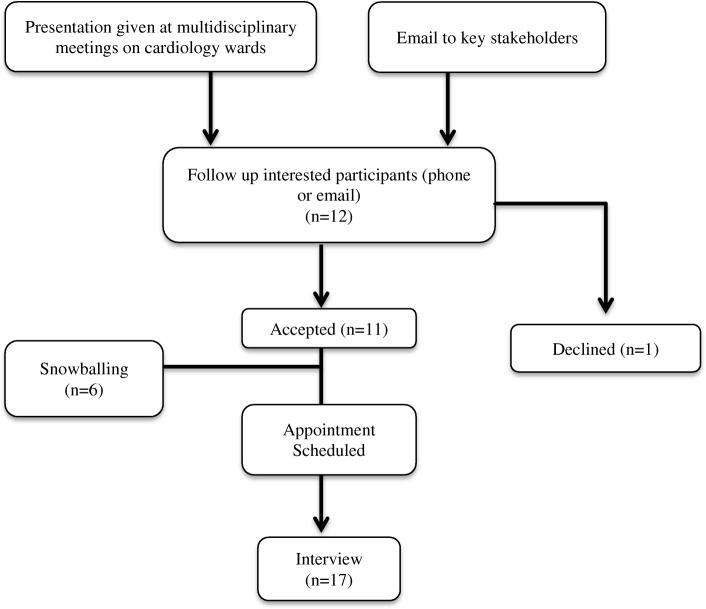
Recruitment of participants

One health professional who originally expressed interest, declined to continue with the study. Non-participation was due to scheduling conflicts. A total of seventeen multidisciplinary staff including 11 nurses, five cardiologists and one Aboriginal health worker, participated in this study (Fig. 1 & Table 1).

**Table 1:** Demographic characteristics of participants (*n* = 17)

Sex	
Males	7
Females	10
Age	
18–24	1
25–34	3
35–44	2
45–54	6
55–64	4
65+	1
Experience working in cardiac related healthcare settings (years)	
< 10	4
10–19	6
20–29	4
30–40	3
Clinical position	
Nursing staff	11
Cardiologist	5
Aboriginal Health Worker	1
Uses supplementary health educational resources^a^ in service and education delivery with patients	
Yes	15
No	2

^a^Supplementary health education materials pre-defined as any printed, audio or audio-visual resources that relay health information, which may include brochures, pamphlets, posters, CDs or DVDs

**Fig. 2:**
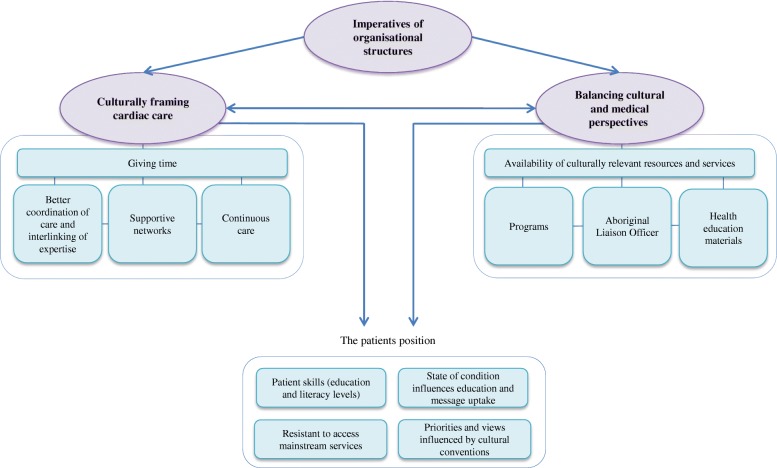
Interrelationships drawn between coded themes

Thematic analysis identified three major overarching themes encompassing: 1) conflicting imperatives of organisational structures; 2) balancing cultural and medical perspectives and, 3) culturally framing cardiac care. The interviewer [JS] drew explicit meaning and identified inter-relationships between these themes through multiple data analysis coding passes. Further sub-themes (*n* = 12) were obtained which represented key concepts provided by participants that influence its superior themes within the hierarchy (Fig. 2).

For the purpose of this report, the three most dominant themes will be the focus of the discussion to enable a more exploratory analysis and address the aims of the current study.

### Theme 1: Conflicting imperatives of organisational structures

Imperatives of organisational structures were noted as the central determinant theme that influenced effective communication and education as indicated by respondents. Even when health professionals felt the desire to deliver education excellence, or there was an expectation of this from management, the imperatives of organisational structures were unsupportive of these efforts and often prompted further deterring barriers. The most common barriers expressed were a lack of time to provide appropriate care to meet the specific needs relevant to patients of the Aboriginal community, as well as very limited availability of culturally appropriate services and resources.

Larger and more complex cultural conventions of the organisation further impacted upon internal priorities of the individual, adding a further challenge. For instance, many participants described experiences when missed appointments, or unexpected visits outside of structured consultations were an issue given stringent pressures around views of other competing job roles requiring prioritisation. This resulted in “limited time” from the perspectives of health professionals to be flexible. For example:

“*Well, I would say in my experience, there’s a very poor attendance rate for scheduled appointments; appointments often have to be rescheduled three, four, five, six times. There is very commonly no communication with my consulting rooms for example when appointments are made if they’re not going to be held so it becomes frustrating for everyone.” (P 10)*

Interestingly, a participant born and raised in a ‘non-westernised’ society, created a link between their own culture and related experiences with Aboriginal Australians. The participant provided a compelling view around the perception that these organisational constructs are inevitably formed by the views and values entrenched within western society.

*“I come from that background where I’m so used to it but still, more you work in a Western society, you like to be one of them. So that’s what happens to us as well so 10, 12 years in same, it’s a monotonous way of following the protocols, so sometimes we need to think beyond protocols...” (P 9)*

The participant supports the idea that not all cultures ‘abide by’ these structures, which is commonly viewed as an ‘unwillingness to comply’ in the eyes of Western society [32]. However, a true understanding of the conflicting priorities and expectations formed by cultural conventions was further explored.

“*His explanations are all very valid explanations why he couldn’t keep that appointment or why he missed those medications because he might have gone to the remote places to attend the family related issues where access is not there for those medications, he ran out of medications – that is not his priority; his priority is to attend the family related or social related issues and the appointment, he wants to make it but something else has come up which is important for him.” (P 9)*

*“If we kind of just remind ourselves that their community have needs that we don't normally meet from a Western point of view.” (P 14)*

Respondents identified that limited use of local health services by patients of the Aboriginal community may be related to a sense of not belonging or not being made to feel welcome within these facilities, thus resulting in disengagement.

*A barrier to Aboriginal people using services is that often there’s no markers to let them know that they are welcome or they are very much part of the population that uses that service, so, to see other Aboriginal people in those circumstances and to, they are perfectly normal, lovely people so I think you know, I should have mentioned that barrier earlier but that obviously meets that – it’s not just about mainstream; it is about everyone’s welcome and you are part of that (P 8).*

*They really do hold off and wait and I’m not really quite sure why but Indigenous Australians included down there, like they really just don't want to see the doctors, don't want to see anyone and then, by the time they come, they’re really quite unwell (P 1).*

### Theme 2: Balancing cultural and medical views

Participants often felt torn and unaware of how to achieve a ‘balance’ in delivering the clinical education that they were responsible to provide, but also acknowledge and empathise with alternative cultural perspectives of health. A lack of culturally specific resources, particularly the availability of Aboriginal-specific education materials, staff and services was emphasised by participants as a barrier to deliver meaningful care.

“*Well, we used to have a Liaison Officer but we don't at the moment. I find it would be nice if we did have one because they sometimes feel more comfortable with someone that’s neutral ground and being able to tap into services in the community that I might not be aware of”. (P 12)*

Participants commented on the value of an Aboriginal Liaison Officer (ALO) as a preeminent resource to facilitate and support education in a hospital-based setting as well as link patients to relevant community-based programs. Of all the participants, only two could identify additional Aboriginal specific educational materials available within their practice. Culturally specific resources are not always well known and hence perhaps are not properly utilised.

When exploring the acceptability and value of the Better Cardiac Care videos for use in clinical practice, all participants agreed that these videos were a valuable addition to current practice. All subjects reported that they believed these resources would support change; although, the reported degree of change attributed to the viewing of these videos varied among responses.

*“I think in the initial phase, yes, but I think there needs to be ongoing reinforcement otherwise old habits, very hard to break and showing them a video after a procedure while they’re keen and motivated to change is all well and good but once you - and this is for any culture, not just for Indigenous – once people go home and get back into their old routine that compliance and that motivation wanes” (P 4)*

Participants felt that the educational video series designed specifically for patients of Aboriginal descent may form part of redressing the ‘imbalance’ in current practice. The idea of simplistic message delivery was a key strength communicated when reviewing the videos. Participants felt that the videos successfully translated comprehensive ‘medical talk’ into ‘layman terminology’ for the viewer.

‘*“Because we all get wrapped up in our own specialty and we often have jargon that goes with that and we know that different services have their own jargon and we tend to talk in that jargon” (P 13*)

*“It’s a good reminder too for health professionals to talk in plain English as well when you’re talking to patients” (P 5)*

An embedded sense of empowerment provided in positive messaging was an element that was appreciated as part of the design. Keywords such as ‘light’, ‘fun’ and ‘joyful’ were used to describe how the participants viewed the main characters’ health journey as depicted in the video series. Participants related this to be a real driver in evoking a sense of hope for the patient to look beyond the now, remain optimistic and focused on what is to come.

*“I think they would really support it because they take the fear away… So, you know, it’s reassuring to me that if something like this would relieve people’s anxiety about their cardiac journey, and it is a journey, because they get moved from here, you know, they don't just stay in this hospital; they’re moved away from their family, they need that reassurance that everything is going to be okay at the other end as well.” (P 6)*

The use of Aboriginal Australians as the actors were said to give a true ‘voice’ to the health messages in the videos as well as the use of appropriate cultural elements such as colloquial language was positively recognised.

*“The fact that it’s by people they may know or it’s by Aboriginal people so they can really relate to what they’re seeing” (P 11).*

*“Well, it’s using terminology like using the “uncle”, “aunty” that sort of stuff that you hear a lot of Aboriginal Australians using …which I think is very appropriate to Aboriginal Australians” (P1)*

The majority of the comments for improvement related to the first video clip (*Aunty Gloria’s Story: At the Hospital)* where participants raised concern that the illustrated story did not establish realistic expectations of a patient’s health journey and that they fear living through the cardiac experience (heart attack) would be more intimidating than is depicted in the video.

*“The only thing I didn’t like was that with that one doctor in the cardiac cath lab, I didn’t think that it was realistic because to see that, it wasn’t as threatening but there’s so many more people in the cath lab than that and I just worry that it might send the wrong message.” (P 6)*

Furthermore, information regarding post procedural care, such as requiring to lay flat for hours after a groin percutaneous coronary intervention (PCI) and the need to call staff if they experience any symptoms such as pain or bleeding, was something that a few participants felt was missing. Participants identified this as a major flaw that needed to be addressed in order to provide a true representation of cardiac management after an Angiogram has been performed.

*“I think the video could talk a little bit more about the post-catheter care instead of being wheeled straight back. I think it would be useful to say things like “The patient might have to lie still for a few hours but that’s normal” like depending on if they go in the groin or they go in the wrist. … Some people might say “Oh, I can’t lie flat for more than 10 minutes”, which is a problem.” (P 6)*

Timing of education was considered pertinent to the patient’s ability to understanding and absorb information, particularly in the context of cardiac events. For example:

“*Sometimes patients are a bit in shock or can’t take information in so you might have to come at a later time when they’re more open to it” (P 16)*

“*It’s a very highly stressful situation and they don’t often absorb a lot of the information all at once but, you know, it reassures me that if they’ve got a resource that they can take home and look at again later – hopefully, it will reinforce what we’ve told them or refresh what we’ve told them here in hospital.” (P 6)*

Interestingly, participants introduced the idea that the act of giving educational materials or resources in written formats were also to provide personal comfort and assurance that they are satisfying part of their professional duties. Others reported that educational materials are useful as they offer a more structured process to portray medical information. Participants expressed that the structure also assisted in message consistency and allowed for instant two-way feedback between the patient and practitioner, to permit individualisation of recommendations that can be tailored to the individual patient’s needs.

### Theme 3: Culturally framing cardiac care

The need for consistent reinforcement of education, which encompasses continuous care and regular follow-up visits was one strategy to improve current practice offered by participants. Models of care vary across wards and specialisations. A participant discussed the possibility of adopting a model of care from other clinical areas that might align better to the specialist needs of Aboriginal and Torres Strait Islanders.

*“… I think we need to grab that model from obstetrics department where you are pregnant, the process you go through, getting a prenatal class, that class, this class, 101 classes happens which may be very time-consuming but very, very useful.” (P 9)*

Additionally, participants reported that it might be of benefit to run a consistently scheduled in-service at a more culturally safe environment such as Aboriginal Medical Services (AMS), which is already an established channel that provides access to patients, rather than just expecting them to seek mainstream service channels.

Better co-ordination of care and collaboration amongst the multidisciplinary team with varied expertise in given areas relating to cardiac care, was emphasised by one participant. The importance of a patient-centred network was stressed, but also equally important was the need to reiterate the same messages to patients. This integration and consistency was noted to likely enhance communication amongst practitioners themselves regarding a patient’s individual care plan and result in better outcomes for all. The participants stressed the significance of building trusting relationships with their patients as an invaluable strength when engaging with individuals of Aboriginal descent.

*“I think what would help is if the health professionals were kind of all singing the same tune. So if we’re all pretty much on the same message… So that endorsement by maybe the cardiologist, the GP and then the cardio rehab team, you know that always has much more value than just one person doing something and, for the patient who thinks “Oh you all talk – you communicate amongst yourselves…”” (P 5)*

Further, the movement from being just the ‘specialist’ to a ‘facilitator’ in order to improve health was an interesting concept offered. Participants emphasised that this did not mean stepping outside of their relevant expertise, but rather taking time to listen to concerns that may not necessarily relate to their own speciality, and help link the patient with relevant people and services to gain the appropriate required advice.

*“… you are a specialist at the same time you are a family physician for them and they might come up and tell they’ve got a knee pain so a lot of people, we say “Okay, you’re going to need to meet your doctor, your GP and he’ll specialist referral” but in their situation what I do is I go one step extra and say “Okay, what medication you are on for the knee pain” then I call their GP and say “He has got the issue. Maybe you should get some MRI or something. You make an arrangement”” (P 9)*

Further training was said to be highly important for improved patient care. One participant described that despite having compulsory education currently in place as an existing online mini-course ran by the Health Education and Training Institute (HETI) for professional development, this was seen as a ‘chore’. It was suggested that more personalised workshops ran by individuals of Aboriginal and Torres Strait islander descent would likely be more successful.

*“I guess in regards to training, it needs to be specific training, so maybe an in-service with an Aboriginal health worker in regards to the barriers and enablers that they come up against.” (P 2)*

## Discussion

The main findings of this study demonstrated that competing organisational imperatives in clinical cardiac healthcare settings often negatively impact on practitioners’ ability to provide culturally appropriate, patient-centred care for Aboriginals and Torres Strait Islanders. In contrast, breaking beyond these imperatives were considered to support more effective and culturally sensitive communication as well as potential solutions to achieve better health care outcome for patients. Health promotion videos, specifically produced using best practice pedagogy to communicate health information to Indigenous audiences were perceived by cardiac care practitioners to be a useful tool to support traditional modes of education. Simplistic, positive and empowering messaging, as well as the use of Aboriginal actors to provide cultural relevance, were all identified to be positive aspects related to the design of the videos. In particular, the use of real-life community members as actors and use of familiar colloquial language may possibly contribute to the acceptability of the message delivery, as commented by the study participants. This concept is further supported by Kreuter et al. [33] whereby matching health promotion materials to characteristics of the target population (as is done using peripheral approaches) can also enhance the group’s receptivity to and acceptance of messages. Moreover, communication that visually reflects the social and cultural world of the audience is more likely to be perceived as familiar and comfortable [33].

Challenges regarding the translation of ‘medical’ information into everyday language that is relevant, sensitive and meaningful to the target audience, was identified as a particular concern by participants. Professionals tend to use technical terminology because it is precise and familiar, and often because there are no exact equivalent or non-technical words available to offer the same meaning [34]. This is seen as a major contributor to miscommunication, despite sharing the same language. However, particular aspects within the video’s content such as setting realistic expectations of the health journey and clarifying post-procedural care were specific suggestions for improvements to both videos. Participants also saw timing of the education delivery as a crucial determinant that directly influences message uptake. Literature supports that patients find it difficult to process medical information because often they are preoccupied with their symptoms, therefore, newly overwhelming emotions make concentration difficult [34]. Moreover, health professionals are known to sometimes try to communicate more information than patients can process [34]. Therefore, ongoing follow up education is essential.

Existing research supports this by suggesting cardiovascular disease outcomes, including disease prognosis, are reliant on the access to quality follow-up and relevant chronic care services [35]. Aboriginal prevalence and service use is poor, particularly in the cardiac rehabilitation services provided in this LHD, which is reflective of what is known elsewhere [36]. Perspectives of competing personal and family demands were identified as having higher priority than an individual’s own health in some experiences with patients from the Aboriginal community, as has been noted by others [36]. Aboriginal Health Workers (AHWs) fulfil an important role in providing a link between Western medical practices and Aboriginal health views [37]. Unfortunately, the lack of culturally specific resources including AHWs, were notably missed by the hospital’s staff in this study. Further to this, cultures within the organisation contributed to the separation of care into distinct systems, namely hospitals and community care, which often lacked continuity and proper coordination [38]. The desire for all experts to come together and provide consistent messages for reinforcement was seen to be an enabler to drive successful outcomes supported by the responses in this study. A reduction in the complexity of the patient’s referral pathway is also likely to result in better engagement for this community and more likely translate into positive outcomes [36]. In line current with recommendations [35], successful health care delivery models need to recognise the values and beliefs of the population it serves. Respondents in this study purposed that a new model of care is required for Aboriginal Australians in cardiac care practice, whereby simply transferring Western value systems to all aspects of patient care, was no longer considered appropriate. As denoted by Brown and colleagues [39], the lack of adequate training of non-Aboriginal health professionals in cultural competencies has led to mainstream hospitals being daunting and unfriendly environments for many patients of Aboriginal descent. In this study, additional training was identified as critically important to provide the professional with confidence in their approach when accommodating the needs of Aboriginal and Torres Strait Islanders. Participants desired further ‘real’ life training experiences as part of an in-service rather than an online webinar, which was said to potentially result in greater compliance by staff.

In-depth interviews proved to be useful to explore the perspectives of health professionals’ responses of current education practices as well as evaluating the newly designed video series. The systematic process of thematic analysis and the use of a main researcher in this study enabled consistency in the interpretations of the responses provided in the data set. However, limitations to the study included having only one main researcher, involved in the sampling and recruiting methods (i.e. purposive sampling and snow-balling) and may have introduced recruitment bias. Another limitation was the lack of Aboriginal Health Workers representation, despite all attempts in recruitment. Therefore, the perspectives gained may not be representative to all health professionals who work in cardiac healthcare settings. Hence these findings provide some general theoretical explanations [27] that will need to be further researched to verify their applicability to other health professionals in the cardiac team as well as other clinical settings.

## Conclusion

Remodelling of organisational structures is required in order to promote a more culturally-friendly and welcoming environment to encourage Aboriginal and Torres Strait Islander patients to engage with mainstream cardiac care services. Specifically, a model of care that allows health professionals to give their time to provide better coordinated, continuous care to facilitate strong networks between all members of the multidisciplinary team is essential. Culturally relevant resources and services available to patients of Aboriginal and Torres Strait Islander descent needs to be improved within the health services which participated in this study. The educational videos that are purposefully developed using principles that are sensitive to Indigenous views of health offer an additional way in which to overcome some of the barriers to effective patient-practitioner health communication.

### Endnote

^1^In this paper, the term Aboriginal has been used to refer inclusively to Australian individuals of Aboriginal and Torres Strait Islander descent. The use Indigenous is when we are referring to features that are identified across different Indigenous peoples.

## Additional file

Additional file 1: **Item S1.** Semi-structured interview guide. **Item S2.** Demographic Questionnaire. (DOCX 33 kb)

## Abbreviations

ALO: Aboriginal Liaison Officer
AMS: Aboriginal Medical Service
HETI: Health Education and Training Institute
NSW MoH: New South Wales Ministry of Health
