# Supplementary material for: Better cardiac care: health professional’s perspectives of the barriers and enablers of health communication and education with patients of Aboriginal and Torres Strait Islander descent
Source: BMC Health Serv Res. 2019 Feb 7;19:106. doi: 10.1186/s12913-019-3917-4 (PMC6367756; doi:10.1186/s12913-019-3917-4)
Supplement: Supplementary file 1 — Item S1. Semi-structured interview guide. Item S2. Demographic Questionnaire. (DOCX 33 kb) [file 12913_2019_3917_MOESM1_ESM.docx]

**Item S1: Semi-structured interview guide**

**Introduction: (5 minutes)**

Morning/Afternoon *[insert participants name]*. Thank you so much for offering your time to be involved in this study. *[Interviewer to introduce self and role in this study]*. As you know we are conducting this study to investigate the acceptability, usability and value of a series of health education videos to be a part of a broader package to improve cardiac care for Aboriginal and Torres Strait Islanders. Over the next forty minutes I would love to hear about your own experiences, challenges and opportunities for effective education and communication with patients of Aboriginal or Torres Strait Islander descent. I will then show you two of the proposed videos to see what you think, ask some questions and have a chat. I would like to make clear that I want you to feel completely safe to share your opinion and that you should not feel uncomfortable about anything you say. Your opinions will not be identified, nor will your name or identity be used. This is for you, [hands participant information statement, written consent form, and demographics questionnaire]. You may have already read the participant information statement that explains the project we are running, what we will do with the information you provide us, and how we will not identify you. If you wish I will give you another few moments to read it all through and then could you please sign the written consent form and complete the demographic questionnaire.

If you have any questions at any stage, please feel free to ask.

*[Once written consent form is signed and demographic questionnaire is complete, interview can begin]* Let’s get started; the interview will begin with a few questions just to get an idea of your own experiences in health care, more specifically with patients who identify as Aboriginal and/ or of Torres Strait Islander descent.

**Exploratory Questions (10 minutes)**

1. Thinking about health education you might be currently or previously involved in, can you tell me how often you would use health education materials within your practice?

Probes: What types or formats of supplementary health education materials do you use?

- Pamphlets
- Posters
- Print outs

1. What circumstances do you find those education materials useful, or not so useful? Could you give me some examples?

Probes: Can you elaborate on this question by relating to things like?

- Time
- Resources available
- Literacy levels

1. Throughout your employment as a healthcare worker and/or health professional, do you worked with patients of Aboriginal descent, or been exposed to areas relating to Aboriginal Health?

Probes: How often do you care for a patient of Aboriginal or Torres Strait Islander descent? How long (years) have you worked in areas relating to Aboriginal Health.

1. Thinking about patients of Aboriginal or Torres Strait Islander descent specifically, can you tell me about the resources you have available to use?

Probes:

- How does it relate to you and your practice?
- How do you use these resources with patients? Can you provide some past examples?

1. Thinking about when you’re working with patients of Aboriginal or Torres Strait Islander descent, what do you see as the challenges for effective education and communication?

Probes:

- Language
- Cultural differences

1. On the same topic, what do you see as the enablers for effective health education and communication with patients of Aboriginal or Torres Strait Islander descent?

Probes:

- Having family members present
- Engaging an Aboriginal Liaison/ Healthcare worker to also be present
- Actively listening
- Asking the patient what they think would be beneficial in terms of management (having an active role in their health care)
- Building rapport

1. Drawing on your experience and observations, what do you think are some solutions or opportunities to improve effective health communication and education with patients of Aboriginal or Torres Strait Islander descent?

Probes:

- Training

**Evaluation Questions (10 minutes)**

Now that we’ve discussed usual practice and opportunities, if you don’t mind I would like to show you two new resources that aims to improve health literacy and provide a better understanding of treatment options for Aboriginal and/or Torres Strait Islanders. These resources are in the form of videos, which is informative about areas relating to cardiac care. Once we’ve viewed both videos, I will ask a couple of questions relating to each video, and then we can have a general discussion about the appropriateness of these videos if they were implemented into your practice for instance. Let’s start with the first video.

*Video 1 Aunty Gloria’s Story: At the Hospital (Maximum 5 minutes)*

1. Since you’ve watched the video 1: Calling an Ambulance, on Cardiac Catheter lab, what did you like about the educational resource?

Probes:

- What did you think about the content: was it informative enough, easy to understand?
- What did you think about the delivery of the content: was the video format useful to relay information? Were the visuals engaging/ culturally acceptable?

1. What didn’t you like about the educational video?

*Video 2 Aunty Gloria’s Story:* *Keeping your heart health (Maximum 5 minutes)*

1. Since you’ve watched the video on Cardiac Rehabilitation, what did you like about the educational video?

Probes:

- What did you think about the content: was it informative enough, easy to understand?
- What did you think about the delivery of the content: was the video format useful to relay information? Were the visuals engaging/ culturally acceptable?

1. What didn’t you like about the educational video?

**Acceptability/ usability questions (10 minutes)**

1. Have you ever seen or heard of these videos before?

Probes:

- If yes? have you been implementing them in your practice?
- If so, how have you been disseminating these videos in your practice?

1. Thinking about your practice, and the challenges and opportunities you’ve spoke about earlier, can you comment on how you think these videos have a place in your practice?
2. Can you comment on whether these videos have successfully meet or failed to meet the needs and barriers that as you previously mentioned?

Probes:

- Why or why not?
- Could you please provide me with specific examples from each video?
- Could you elaborate by providing examples on what key features made the resources successful or unsuccessful?

1. Now thinking specifically about patients of Aboriginal or Torres Strait Islander descent, how do you think these videos might support or inhibit motivation to change?

Probes:

- Could you elaborate by providing specific examples, with reference to each of the two videos?
- Is/ isn’t acceptable to cultural beliefs
- Does/ doesn’t align with local Aboriginal views
- Does/ doesn’t provide enough information
- Negative or overwhelming messages

1. Again thinking about your experiences in your practice, where would you like to see these videos being shown to patients of Aboriginal or Torres Strait Islander descent, for it to be most beneficial?

Probes:

- Could you elaborate by providing specific examples, with reference to each of the two videos?
- At home (given as a DVD)
- In the hospital: Prior to admission, Post admission
- In the community (available at Aboriginal medical services)

1. Relating to the previous question, who do you think is the best person to provide access to these videos?

Probes:

- Why them?
- Why not (* whoever they don’t mention e.g. nurses, Aboriginal health care workers, Cardiologists)?

1. Finally, would you like to comment how these videos might be improved?

Probes:

- Content
- Quality
- Additional resources (supportive materials)
- Acceptability
- Convenience

[End of interview]

**Thank you so much for your time and contribution, it was lovely to meet you.**

**Item S2: Demographic Questionnaire**

Thank you for expressing interest in this study. Please complete the information below:

1. Sex (please tick)

- Male
- Female

2. Age (please tick)

- 18-24 years old
- 25-34 years old
- 35-44 years old
- 45-54 years old
- 55-64 years old
- 65 years or older

3. How many years of experience do you have working in healthcare settings relating to cardiac care (i.e. hospital settings, community rehabilitation services)?

______________________________________________________________________________________________________________________________________________________

4. Please state your job position in NSW Hospital

______________________________________________________________________________________________________________________________________________________

5. Do you use supplementary health education materials to assist your patient education delivery in your practice? Supplementary health education materials pre-defined as any printed, audio or audio-visual resources that relay health information, which may include brochures, pamphlets, posters, CDs or DVDs. (Please tick correct answer)

- Yes
- No

If yes, please elaborate how often you would use supplementary health education materials in practice? ______________________________________________________________________________________________________________________________________________________
